# The pro-metastasis tyrosine phosphatase, PRL-3 (*PTP4A3*), is a novel mediator of oncogenic function of BCR-ABL in human chronic myeloid leukemia

**DOI:** 10.1186/1476-4598-11-72

**Published:** 2012-09-21

**Authors:** Jianbiao Zhou, Lip-Lee Cheong, Shaw-Cheng Liu, Phyllis SY Chong, Sylvia Mahara, Chonglei Bi, Kelly OK Ong, Qi Zeng, Wee Joo Chng

**Affiliations:** 1 Cancer Science Institute of Singapore, Singapore, Singapore; 2 Department of Medicine, Yong Loo Lin School of Medicine, National University of Singapore, Singapore, Singapore; 3 Department of Hematology-Oncology, National University Hospital, Singapore, Singapore; 4 Institute of Molecular and Cell Biology (IMCB), A*Star, Singapore, Singapore

**Keywords:** Chronic myeloid leukemia (CML), Protein-tyrosine phosphatase of regenerating liver 3 (PRL-3), PTP4A3, BCR-ABL, Imatinib, Tyrosine kinase inhibitor (TKI)

## Abstract

**Background:**

Resistance to tyrosine kinase inhibitors (TKIs) remains a challenge in management of patients with chronic myeloid leukemia (CML). A better understanding of the BCR-ABL signalling network may lead to better therapy.

**Findings:**

Here we report the discovery of a novel downstream target of BCR-ABL signalling, PRL-3 (*PTP4A3*), an oncogenic tyrosine phosphatase. Analysis of CML cancer cell lines and CML patient samples reveals the upregulation of PRL-3. Inhibition of BCR-ABL signalling either by Imatinib or by RNAi silencing BCR-ABL reduces PRL-3 and increases cleavage of PARP. In contrast, the amount of PRL-3 protein remains constant or even increased in response to Imatinib treatment in drug resistant cells expressing P210 T315I. Finally, analysis with specific shRNA shows PRL-3 involvement in the proliferation and self-renewal of CML cells.

**Conclusions:**

These data support a role for PRL-3 in BCR-ABL signalling and CML biology and may be a potential therapeutic target downstream of BCR-ABL in TKI resistant mutant cells.

## Findings

Chronic myeloid leukemia (CML) is a hematopoietic stem cell malignancy with a hallmark cytogenetic abnormality, i.e., the *BCR-ABL* fusion oncogene, resulting from the reciprocal translocation of chromosomes 9 and 22 [also known as Philadelphia (Ph) chromosome] [1]. CML is the best and most successful disease model for tyrosine kinase inhibitor (TKI) therapy [2,3]. Unfortunately, acquired resistance can develop during the course of treatment. Effective therapies that can overcome resistance still remain a challenge for the clinical management of CML [2,4]. The mechanism of BCR-ABL induced transformation and signaling transduction networks have been intensively characterized over the decades [5-7]. However, new discoveries related to the BCR-ABL signaling pathway and mechanisms of TKI resistance continues to emerge, leading to a better understanding of disease progression and development of novel therapy [8-10].

Protein-tyrosine phosphatase of regenerating liver 3 (PRL-3, encoded by *protein tyrosine phosphatase type IVA 3*, *PTP4A3*) belongs to class I cysteine-based protein tyrosine phosphatases (PTPs) with dual-specificity [11-13]. PRL-3 has been identified as a critical player in cancer cell metastasis, invasion, migration, and tumor angiogenesis [11,14-16]. The association between elevated PRL-3 and the development of various human cancers has been validated in a wide range of solid tumors [11,14,15] and multiple myeloma [17].

We recently discovered that poly(rC) binding protein 1 (PCPB1, also known as heterogenous nuclear ribonucleoprotein E1, hnRNP-E1) inhibited PRL-3 protein through binding 5’-UTR (untranslated region) of *PRL-3* mRNA [18] and showed that PRL-3, acting as a downstream target of the internal tandem duplication (ITD) of fms-like tyrosine kinase (FLT3) signaling, was implicated in FLT3 inhibitor therapy in acute myeloid leukemia (AML) [19]. Furthermore, PRL-3 also has been demonstrated as an independent prognostic parameter for poor overall survival (OS) and event-free survival (EFS) in AML [20]. Importantly, targeting intracellular PRL-3 protein suppressed cancer growth [21]. In the present study, we hypothesize that PRL-3 might be involved in leukemogenesis of human CML.

## Overexprsesion of PRL-3 in CML cell lines and primary patient samples

**Figure 1:**
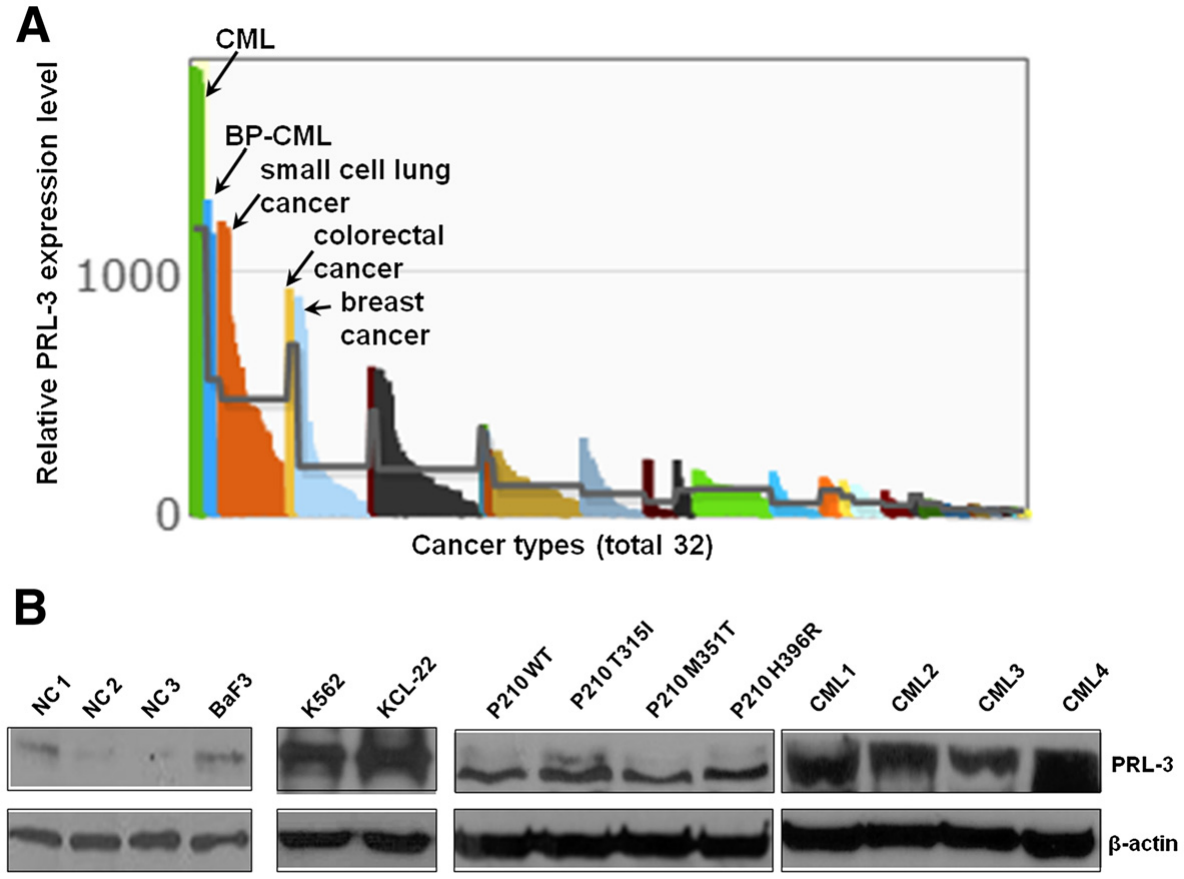
**PRL-3 expression in CML cell lines and primary CML bone marrow cells.** (**A**) The relative expression level of PRL-3 in a gene expression database (E-MTAB-37). The top five cancers with the highest PRL-3 transcript were indicated as CML, BP (blast phase)-CML, small cell lung cancer, colorectal cancer, and breast cancer. The complete list of the different cancer types is available on European Bioinformatics Institute (EBI) website. (**B**) Analysis of PRL-3 protein in normal control (NC) BM samples, untransfected parental BaF3 cells, CML cell lines and primary CML BM samples (CML1, 2, 3, 4) by Western blot. β-actin was used as loading control. More details were described in Additional file 1.

A search of the Gene Expression Atlas (http://www.ebi.ac.uk/gxa/gene/ ENSG00000184489) showed that the expression level of *PRL-3* was highest in CML among 950 human cancer cell lines covering 32 different types of cancers (Dataset code: E-MTAB-37), suggesting a potential role of PRL-3 in CML pathogenesis (Figure 1A). To further confirm PRL-3 expression, we examined PRL-3 protein levels in a panel of CML cell lines and primary CML BM samples. By immunoblot analysis,(Additional file 1) we observed strong PRL-3 protein expression in two human CML cell lines (K562 and KCL-22, Figure 1B), murine hematopoitic cells expressing WT and mutant BCR-ABL constructs (P210 WT, P210 T315I, P210 M351T and P210 H396R, Figure 1B middle), and primary BM samples from CML patients (Figure 1B right). It is worth noting that PRL-3 is either not expressed or minimally expressed in bone marrow cells from 3 normal controls (NC) or parental BaF3 cells (Figure 1B) [19]. Altogether, our data obtained from Western blot analysis of CML cell lines and primary CML samples, as well as the analysis of a publicly available gene expression dataset demonstrated over-expression of PRL-3 in CML.

## Imatinib suppressed PRL-3 through inhibition of STAT pathway

**Figure 2:**
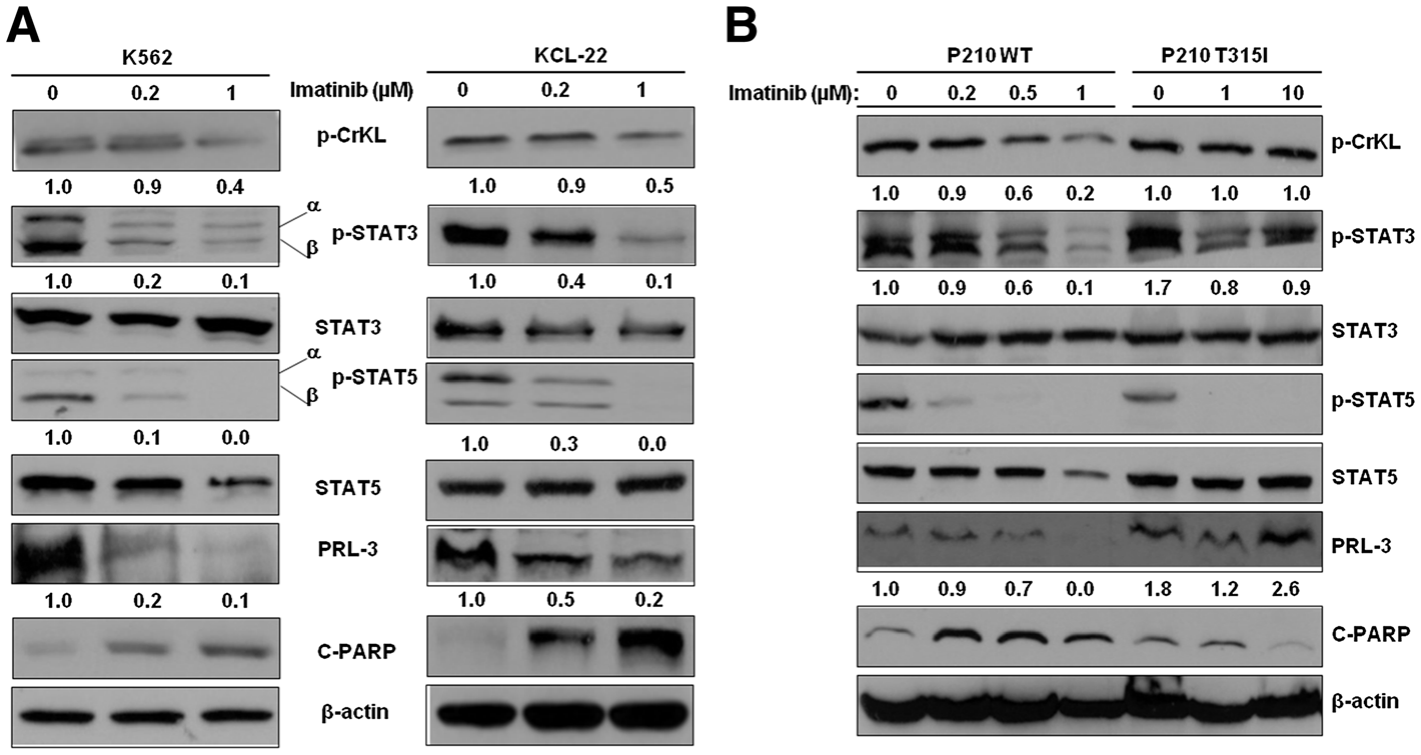
**Involvement of PRL-3 in CML response to Imatinib and CML proliferation and self-renewal.** (**A**) K562 and KCL-22 cells were treated with Imatinib 0 (vehicle control), 0.2 and 1 μM for 48 h. Cells were harvested and followed by Western blot analysis of a panel of proteins shown. (**B**) P210 WT and P210 T315I cells were treated with either vehicle control or various concentrations of Imatinib as indicated for 48 h. Cell lysates were used for Western blot analysis of proteins as indicated. In both (**A**) and (**B**), β-actin was used as a loading control. Densitometric analysis was performed using Amersham Image Scanner with LabScan ImageQuant TL Software.

Imatinib blocks the binding of ATP to the BCR-ABL tyrosine kinase [22,23], and is currently used as the first-line treatment for CML [2,4]. To establish a connection between BCR-ABL signalling and PRL-3 expression, we treated human CML cell lines, K562 and KCL-22 cells with Imatinib and assessed the expression of PRL-3. Western blot analysis demonstrated that Imatinib dose-dependently decreased p-CrkL (a surrogate marker of BCR-ABL kinase activity), p-STAT3, p-STAT5, as well as PRL-3 (Figure 2A). Consistent with the effective inhibition of oncogenic BCR-ABL signalling, cleaved-PARP, a hallmark of apoptosis was increased as a response to the Imatinib treatment (Figure 2A). We next tested whether Imatinib could induce PRL-3 protein down-regulation in BaF3 murine hematopoietic cells engineered to express either wild-type, or the Imatinib resistant T315I mutant P210 BCR-ABL. As expected, the expression of p-CrkL, p-STAT3 and PRL-3 was down-regulated in a dose-dependent manner in the imatinib sensitive P210 WT cells. In contrast, BCR-ABL activity in P210 T315I cells was resistant to Imatinib even at high doses (10 μM) as indicated by no change in p-CrkL. In this resistant cell line, PRL-3 was not downregulated but rather its level increased at higher doses of Imatinib (Figure 2B). Surprisingly, p-STAT5 expression was almost completely abolished in both P210 WT and p210 T315I cells upon exposure to Imatinib (Figure 2B). On the other hand, the down-regulation of PRL-3 correlated with the inhibition of STAT3. Consistent with inhibition of BCR-ABL, increased PARP cleavage fragment was observed in cells sensitive to Imatinib (K652, P210 WT), but not in resistant cells (P210 T315I) (Figure 2B). In addition to the STAT pathways, PI3K/AKT and MAPK/ERK signalling pathways were also downstream of BCR-ABL signalling and may contribute to the transformation of CML cells [7]. However, inhibition of these two pathways did not correlate with the down-regulation of PRL-3 protein in the P210 and K562 cells (Additional file 2). Taken together, these data suggest PRL-3 is downstream of BCR-ABL mainly through the STAT pathway in CML.

## Silencing BCR-ABL fusion gene or STAT3 decreased PRL-3 expression

**Figure 3:**
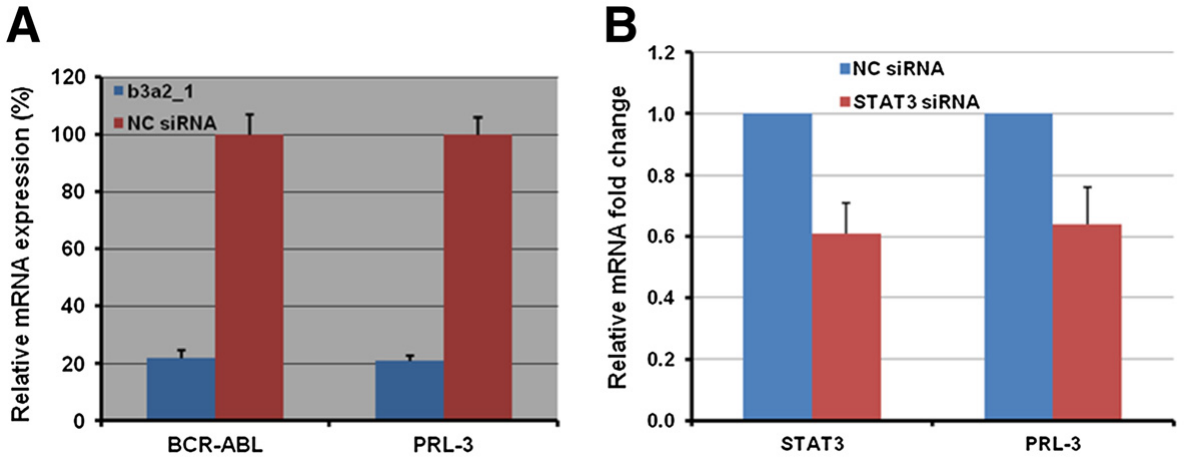
**Effect of silencing *BCR-ABL *fusion gene and *STAT3 *on *PRL-3 *expression.** (**A**) Quantification of *BCR-ABL* and *PRL-3* mRNA by qRT-PCR in K562 cells transfected with b3a2_1 siRNA and non-targeting control (NC) siRNA. Two million cells were nucleofected with 30 nM b3a2_1 siRNA or NC siRNA together with pmaxGFP (Lonza, Switzerland) as an indicator of transfection efficacy. The solution V and program T-016 were used as recommended by the manufacturer (Lonza). The transfection efficacy was about 80%. RNAs were extracted 24 h after transfection. Primer sequences, siRNA sequences and qRT-PCR methods were described as Scherr *et al.*[24] and Zhou *et al.*[19]. (**B**) qRT-PCR quantification of *STAT3* and *PRL-3* gene expression in K562 cells transfected with STAT3 siRNA (Santa Cruz Biotechnologies, Inc., CA, USA) and NC siRNA in a Nucleofection device as described in (**A**). The primer sequences of STAT3 are as following: 5’-AGGATGGCCCAATGGAATCAGCTA-3’ (sense) and 5’-AGCGGCTATA CTGCTGGTCAATCT-3 (antisense).

To further confirm that the downregulation of PRL-3 is not due to off-target effect of Imatinib, we used small interfering RNA (siRNA) to specifically knock down the *BCR-ABL* fusion gene as reported by Scherr *et al*[24]. qRT-PCR analysis confirmed the expression of PRL-3 gene was decreased approximate 80% in b3a2_1 siRNA transfected K562 cells, in parallel with reduction of *BCR-ABL* fusion genes (Figure 3A) confirming that PRL-3 is downstream of BCR-ABL signalling. To assess the specific role of STAT3 in upstream regulation of PRL-3, we decreased *STAT3* mRNA by using siRNA in a Nucelofection device. qRT-PCR showed that the expression of *PRL-3* was reduced in STAT3 siRNA expressing K562 cells compared with the control (NC) siRNA expressing K562 cells (Figure 3B). These results support the BCR-ABL and STAT3 are specific upstream regulators of PRL-3 signalling.

## PRL-3 is involved in CML proliferation, self-renewal, tumorigenic capacity and drug response

**Figure 4:**
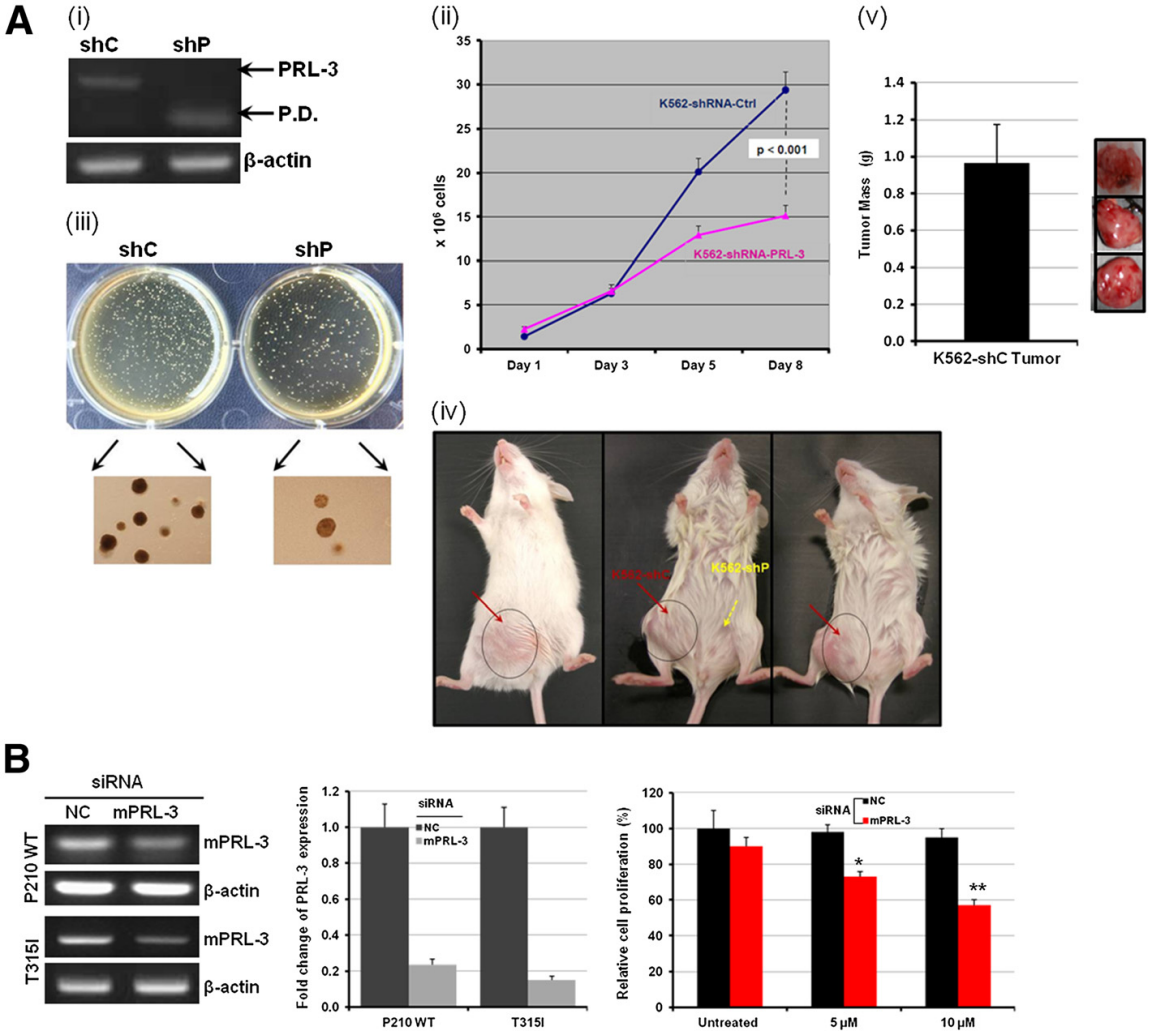
**Functional consequences of silencing *PRL-3 *in CML cells.** (**A**) The mRNA expressions of *PRL-3* and *β-actin* were analyzed RT-PCR in K562-shRNA-Scramble (shC) and K562-shRNA-PRL-3 (shP) transduced cells (i). P.D. stands for primer dimer (i). Cell proliferation curves were constructed by the total viable cells in ShC- and ShP-K562 cells cultured over 8 days. Cell proliferation and viability were determined by trypan blue counting in every other day. Results represent the mean ± SD of triplicates (ii). Colony Forming Unit (CFU) assay of in ShC- and ShP-K562 cells. The experiments were duplicated. The upper panel of images was taken by a Canon EOS40D camera and the lower panel of pictures was captured in 4 × 10 magnification field under an invert microscopy. The experiments were duplicated and representative pictures were presented (iii). (iv) Comparison of tumor formation capacity in mouse xenograft models between K562-shC and K562-shP cells. (v) Average tumor weight of three K562-shC tumors. Bar indicates standard deviation (SD). (**B**) P210 WT and T315I cells were nucleofected using the cell line solution V (Lonza), program X-001 and nontargeting (NC) or mouse PRL-3 siRNA (Santa Cruz Biotechnology, Inc.). After 24 h, cells were subjected to conventional RT-PCR and qRT-PCR analysis of PRL-3 expression or MTS assays. Cell proliferation assessments in untreated, Imatinib-treated (5, 10 μM) P210 T315I cells transfected with either nontargeting (NC) or mouse PRL-3 siRNA for 48 h and determined by MTS assay. Results were presented the mean ± SD of 3 independent experiments. Significant value *p < 0.05 and **p < 0.01.

To assess the functional effect of PRL-3 in CML, we knocked down PRL-3 using short-hairpin RNA (shRNA). RT-PCR analysis showed that shRNA-PRL-3 transduced K562 cells (K562-shP) demonstrated significantly reduced *PRL-3* mRNA levels as compared to shRNA-scramble control transduced cells (K562-shC) (Figure 4Ai). K562-shP cells proliferated as much as 2-fold slower than K562-shC at day 8 (p < 0.001) (Figure 4Aii). K562-shP cells also showed significantly impaired colony generating capacity, an indicator of self-renewal capacity, by 3-fold compared to K562-shC (p < 0.001) (Figure 3Aiii). We next evaluated the oncogenic role of PRL-3 in CML in vivo. Three million of K562-shC and K562-shP cells were subcutaneously inoculated into the right and left side of NOD/SCID mice, respectively (3 mice total). After 3 weeks, only K562-shC cells developed tumors at average size 800 mm^3^ (Figure 4Aiv, right side of the animals, indicated by black circles) and average tumor weight was 0.967 ± 0.21 g (Figure 4Av). These results indicate a critical role for PRL-3 in CML cell expansion, and self-renewal *in vitro* and *in vivo*. To examine if PRL-3 could be a potential therapeutic target downstream of BCR-ABL especially in TKI resistant cells with BCR-ABL mutations, we utilized RNAi to knock down PRL-3 expression in P210 T315I cells. Downregulation of mouse PRL-3 (mPRL-3) was confirmed by RT-PCR (Figure 4B, left panel) and qRT-PCR (Figure 4B, middle panel). Indeed, while Imatinib had no effect on P210 T315I cells, mPRL-3 silencing led to significant cell death in these cells (Figure 4B, right panel).

In summary, the present study demonstrates that PRL-3 is upregulated in human CML cell lines, BCR-ABL transformed cell lines and primary CML patient samples. Interestingly, in a previous study, high expression of PRL-3 has been associated with aggressive phenotype of *BCR-ABL* positive acute lymphoblastic leukemia (ALL) [25]. This finding, together with our results highlight that PRL-3 is a novel downstream target of the BCR-ABL signalling pathway, and may be a novel mediator of BCR-ABL oncogenic functions such as cell survival and self-renewal. Suppression of PRL-3 could provide potential opportunity for further improving anti-CML therapy, especially in tumors with Imatinib or TKI resistant BCR-ABL mutants.

## Competing interests

The authors declare no conflict of interests.

## Authors’ contributions

JZ, WJC conceptualized the original idea, designed the experiments and analyzed the data. JZ performed the experiments, wrote the paper. LLC, SCL, PSYC, SM, CB, KOKO contributed to the experiments. QZ provided critical reagents and contributed to discussions and proofread the manuscript. All authors read and approved the final manuscript.

## Supplementary Material

Additional file 1 Supplementary Methods. Click here for file

Additional file 2 **Figure Inhibition of MAPK/ERK and PI3K/AKT pathway activities was not correlated to down-regulation of PRL-3 protein level.** (A) P210 WT and P210 T315I cells were treated with either vehicle control or various concentrations of Imatinib as indicated for 48 h. Cell lysates were used for Western blot analysis of proteins as indicated. (B) K562 cells were treated with Imatinib 0 (vehicle control), 0.2 and 1 μM for 48 h. Cells were harvested and followed by Western blot analysis of a panel of proteins shown. In both (A) and (B), β-actin was used as a loading control. MOLM-14 cell lysates were used as positive controls for p-AKT antibody. Densitometric analysis was performed using Amersham Image Scanner with LabScan ImageQuant TL Software. Click here for file
