# Supplementary material for: The pro-metastasis tyrosine phosphatase, PRL-3 (PTP4A3), is a novel mediator of oncogenic function of BCR-ABL in human chronic myeloid leukemia
Source: Mol Cancer. 2012 Sep 21;11:72. doi: 10.1186/1476-4598-11-72 (PMC3537646; doi:10.1186/1476-4598-11-72)
Supplement: Additional file 1 — Supplementary Methods. [file 1476-4598-11-72-S1.doc]

**Supplementary Methods**

**1. Cell culture and patient samples**

The culture of CML cell lines, including K562, KCL-22, BaF3, BaF3/BCR-ABL-WT (P210 WT), BaF3/BCR-ABL-T315I (P210 T315I), BaF3/BCR-ABL-M351T (P210 M351T) and BaF3/BCR-ABL-H396R (P210 H396R) cells were cultured with RPMI1640 (Invitrogen) supplemented with10% of fetal bovine serum (FBS, JRH Bioscience Inc, Lenexa, KS) at density of 2 to 10 x 10^5^ cells/ml in a humid incubator with 5% CO_2_ at 37ºC. Bone marrow samples from newly diagnosed and previously untreated CML patients in the presence of Ph chromosome or *BCR-ABL* fusion gene in chronic phase (<15% blasts in peripheral blood and bone marrow) were obtained at National University Hospital (NUH) in Singapore following informed consent. This study was approved by Institutional Review Board (IRB) of National University of Singapore and BM samples were obtained with written informed consent.

**2. Drug preparation**

Imatinib was obtained from Novartis and dissolved in 1 x PBS as 10 mM stock.

**3. Cell viability assays**

Leukemic cells were seeded in 96-well culture plates at a density of 2 × 10^4^ viable cells/100 µl/well in triplicates, and were treated with Imatinib. Colorimetric CellTiter 96 AQ_ueous_ One Solution Cell Proliferation Assay (MTS assay, Promega, Madison, WI) was used to determine the cytotoxicity. Each experiment was in triplicate.

**4. Western blot**

Preparation of the cell lysate and immunoblotting were performed as previously described in reference 12 (Zhou J, *et al*., 2011),^21^ except for PRL-3 analysis, when 16% gel was used. Anti-PRL3 antibody was clone 318 as reported previously (Zhou J, *et al*., 2011). Antibodies used were as follows: anti-STAT3, anti-p-STAT3 (Tyr705), anti-STAT5, anti-p-STAT5 (Tyr694), anti-AKT, anti-p-AKT (Ser473),, anti-ERK1/2, anti-p-ERK1/2 (Thr202/Tyr204), anti-cleaved poly (ADP-ribose) polymerase (PARP), from Cell Signaling Technology (CST, Danvers, MA) and anti-Actin from Santa Cruz Biotechnology (Santa Cruz, CA).

**5.** **Short-hairpin (shRNA) RNA study**

Control and 3 human PRL-3 specifically pLKO.1 lentiviral shRNAs (RHS3939-9570404, -98490774 and -98490780) were purchased from Open Biosystems (Huntsville, AL, USA). Three million of K562 cells were mixed with viral supernatant and 8 μg/ml of polybrene (Millipore) and centrifuged at 2500 rpm for 90 min at 30ºC. After additional incubation at 37ºC for 4 hours, the medium was changed to fresh complete medium. Two days later, cells were submitted to cell growth assay, colony forming unit (CFU) assay or RNA extraction, followed by RT-PCR analysis of PRL-3 expression.

**6. Nucleofection of leukemic cells**

Knockdown of *BCR-ABL* fusion gene in K562 using electroporation with nontargetting control siRNA or siRNA b3a2_1 method was carried out in a Amaxa Nucleofection device with Kit V and Program T-016 as recommended by the manufacturer (Lonza, Cologne, Germany). The sequence of siRNA b3a2_1 was described in reference 15. Silencing of PRL-3 in P210 T315I cells was nucleofected with Kit V and Program T-001. siRNA targeting murine PRL-3 was purchased from Santa Cruz Biotechnology (Santa Cruz, CA).

**7. RT-PCR and real-time quantitative (q)-RT-PCR**

Reverse-transcription polymerase chain reaction (RT-PCR) and qRT-PCR were also performed as published in reference 12 and 15.

**8.** **Colony forming unit (CFU) assay**

Control shRNA transduced or PRL-3 shRNA transduced K562 cells were washed twice with 1 x PBS. After that, about 3000 cells each were plated in MACS® HSC-CFU media complete with Epo (Miltenyi Biotec GmbH, Germany) in 6-well plates and cultured for 7 days at 37ºC in a 5% CO_2_ incubator. Colonies consisting of more than 50 cells were counted under an inverted microscope. Total 5 random 4 x 10 magnification fields were selected and average number of colonies of each sample was calculated. The experiments were duplicated.

**9.** **Xenograft mouse model**

Female non-obese diabetic severe combined immunodeficiency (NOD/SCID) mice (17-20 g, 4-6 weeks old) were bred in house (kindly provided Dr. Shing-Leng Chan, Cancer Science Institute of Singapore). Three million of control shRNA transduced or PRL-3 shRNA transduced K562 cells were washed twice with 1 x PBS and were subcutaneously injected into loose skin between the lower abdomen and right hind leg and left hind leg of recipient mice, respectively. The experiment was comprised of 3 mice.

The length (L) and width (W) of the tumor were measured with callipers, and tumor volume (TV) was calculated as TV = (L×W^2^)/2. The protocol was reviewed and approved by Institutional Animal Care and Use Committee in compliance to the guidelines on the care and use of animals for scientific purpose.

**10. Statistical analysis**

Student’s t-test was employed to compare the difference between control and experimental group and p values of < 0.05 were considered to be significant.
