# Supplementary material for: The pro-metastasis tyrosine phosphatase, PRL-3 (PTP4A3), is a novel mediator of oncogenic function of BCR-ABL in human chronic myeloid leukemia
Source: Mol Cancer. 2012 Sep 21;11:72. doi: 10.1186/1476-4598-11-72 (PMC3537646; doi:10.1186/1476-4598-11-72)
Supplement: Additional file 2 — Figure Inhibition of MAPK/ERK and PI3K/AKT pathway activities was not correlated to down-regulation of PRL-3 protein level. (A) P210 WT and P210 T315I cells were treated with either vehicle control or various concentrations of Imatinib as indicated for 48 h. Cell lysates were used for Western blot analysis of proteins as indicated. (B) K562 cells were treated with Imatinib 0 (vehicle control), 0.2 and 1 μM for 48 h. Cells were harvested and followed by Western blot analysis of a panel of proteins shown. In both (A) and (B), β-actin was used as a loading control. MOLM-14 cell lysates were used as positive controls for p-AKT antibody. Densitometric analysis was performed using Amersham Image Scanner with LabScan ImageQuant TL Software. [file 1476-4598-11-72-S2.doc]

**Supplementary Figure**


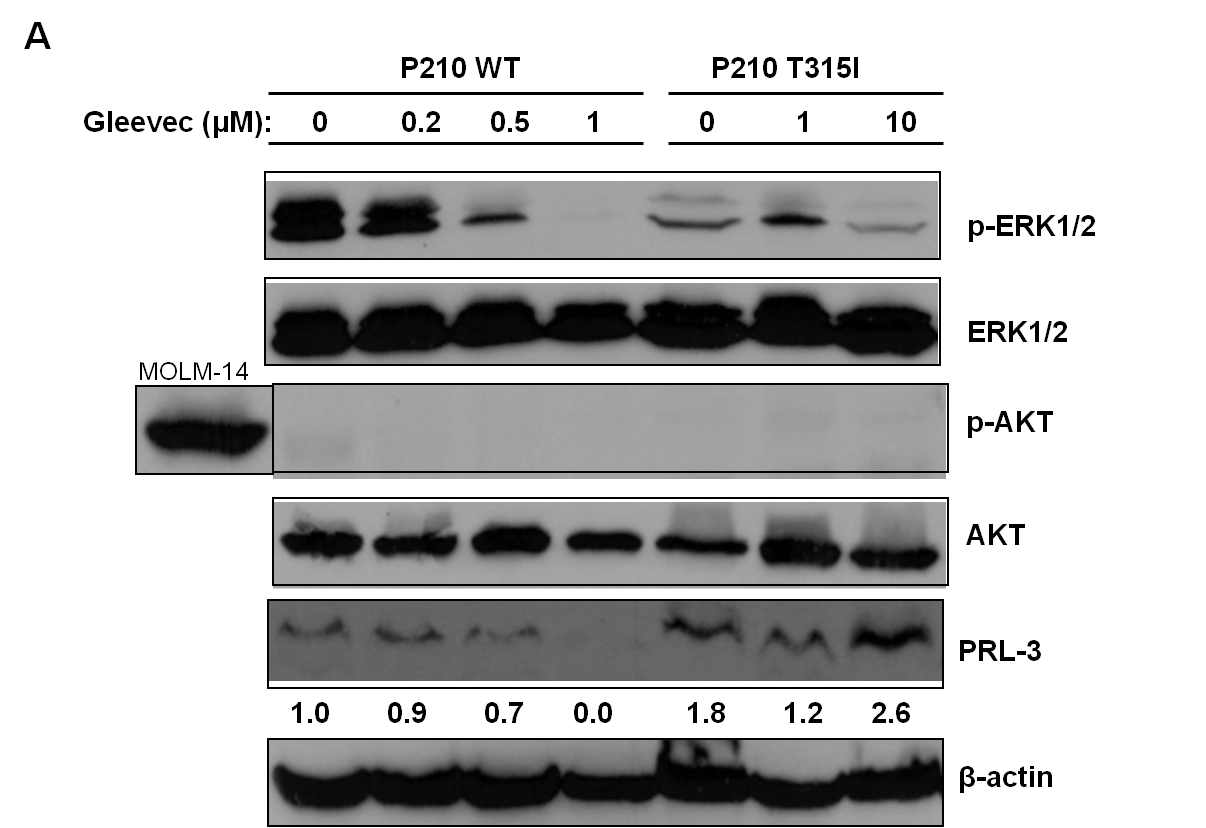

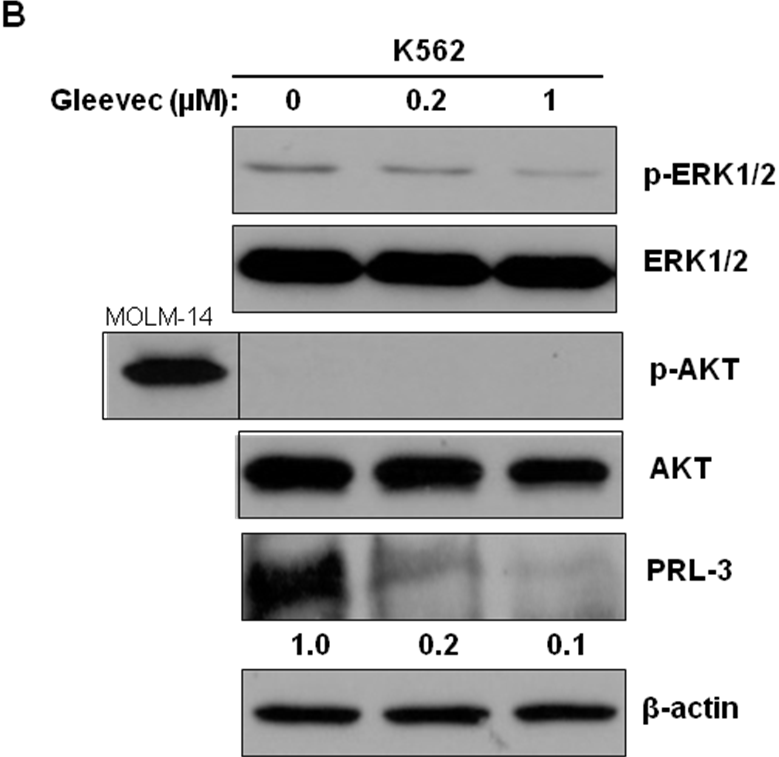


**Supplementary Figure. Inhibition of MAPK/ERK and PI3K/AKT pathway activities was not correlated to down-regulation of PRL-3 protein level.** (A) P210 WT and P210 T315I cells were treated with either vehicle control or various concentrations of Imatinib as indicated for 48 hours. Cell lysates were used for Western blot analysis of proteins as indicated. (B) K562 cells were treated with Imatinib 0 (vehicle control), 0.2 and 1 μM for 48 hours. Cells were harvested and followed by Western blot analysis of a panel of proteins shown. In both (A) and (B), β-actin was used as a loading control. MOLM-14 cell lysates were used as positive controls for p-AKT antibody. Densitometric analysis was performed using Amersham Image Scanner with LabScan ImageQuant TL Software.
